# Histone deacetylase inhibition accelerates the early events of stem cell differentiation: transcriptomic and epigenetic analysis

**DOI:** 10.1186/gb-2008-9-4-r65

**Published:** 2008-04-04

**Authors:** Efthimia Karantzali, Herbert Schulz, Oliver Hummel, Norbert Hubner, AK Hatzopoulos, Androniki Kretsovali

**Affiliations:** 1 Institute of Molecular Biology and Biotehnology, FORTH, Heraklion 71110 Greece; 2 Department of Biology, University of Crete, Heraklion 71409 Greece; 3 Max-Delbruck-Center for Molecular Medicine-MDC, Berlin 13092, Germany; 4 GSF, Institute of Clinical Molecular Biology and Tumor Genetics, 81377 Munich, Germany; 5 Vanderbilt University, Department of Medicine - Division of Cardiovascular Medicine and Department of Cell and Developmental Biology, Nashville Nashville, TN 37232-2358, USA

## Abstract

**Background:**

Epigenetic mechanisms regulate gene expression patterns affecting cell function and differentiation. In this report, we examine the role of histone acetylation in gene expression regulation in mouse embryonic stem cells employing transcriptomic and epigenetic analysis.

**Results:**

Embryonic stem cells treated with the histone deacetylase inhibitor Trichostatin A (TSA), undergo morphological and gene expression changes indicative of differentiation. Gene profiling utilizing Affymetrix microarrays revealed the suppression of important pluripotency factors, including Nanog, a master regulator of stem cell identity, and the activation of differentiation-related genes. Transcriptional and epigenetic changes induced after 6-12 hours of TSA treatment mimic those that appear during embryoid body differentiation. We show here that the early steps of stem cell differentiation are marked by the enhancement of bulk activatory histone modifications. At the individual gene level, we found that transcriptional reprogramming triggered by histone deacetylase inhibition correlates with rapid changes in activating K4 trimethylation and repressive K27 trimethylation of histone H3. The establishment of H3K27 trimethylation is required for stable gene suppression whereas in its absence, genes can be reactivated upon TSA removal.

**Conclusion:**

Our data suggest that inhibition of histone deacetylases accelerates the early events of differentiation by regulating the expression of pluripotency- and differentiation-associated genes in an opposite manner. This analysis provides information about genes that are important for embryonic stem cell function and the epigenetic mechanisms that regulate their expression.

## Abstract 2

A gene profiling study of mouse embryonic stem cells treated with the histone deacetylase inhibitor trichostatin A shows that inhibition of histone deacetylases accelerates the early events of differentiation, by regulating the expression of pluripotency- and differentiation-associated in an opposite manner.

## Background

Embryonic stem (ES) cells have attracted intense interest because they offer great promise for tissue regeneration in cell-based therapies. In addition, they provide an excellent experimental system to study development and differentiation using *in vivo *and *in vitro *strategies.

ES cells can be cultivated *in vitro *while retaining their undifferentiated character and self-renewing capacity [1,2]. Signal transduction mechanisms implicated in self-renewal are the LIF/Stat3 pathway for murine ES cells [3], and bone morphogenetic protein [4] and the Wnt pathway [5] for both mouse and human stem cells. Intrinsic factors that maintain self-renewal include the transactivators Oct4, Sox2 and Nanog [1]. The three transcription factors form a regulatory circuit that has auto- and cross-regulatory activities [6] and is associated with both active and silenced genes [6,7]. This initial 'stemness core' has been recently extended by the addition of Klf4 [8] and Sall4 [9]. Moreover, novel factors that contribute to pluripotency have been identified using an RNA interference approach [10] or Nanog affinity co-purification strategies [11]. These new discoveries suggest that regulation of stemness may be far more complex than previously thought.

Superimposed on this genetic program, epigenetic mechanisms may also determine the composition of the stem cell transcriptome. Post-translational modifications of histones are indicative of chromatin structure and regulate gene activation and repression during development [12,13]. For example, lysine acetylation of various residues on histone H3 and H4 and lysine methylations of H3K4, H3K36 and H3K79 are involved in transcriptional activation whereas methylation of H3K9, H3K27 and H4K20 are linked to transcriptional silencing [14]. The chromatin of ES cells has a characteristic structure of increased accessibility compared to differentiated cells, due to fewer and loosely bound histones and architectural proteins [15]. Trimethylation of K4 and K27, mediated by Trithorax and Polycomb groups, respectively, have important functions in the determination of stem cell state and differentiation commitment [16,17]. Lineage-specific genes, which are silenced in the undifferentiated state by polycomb complexes [18,19], are 'bivalently' marked with both modifications [16,17,20-22]. This mark is considered a means of keeping developmental genes poised for rapid activation during stem cell differentiation [20,21], although it is neither a unique feature of ES cells [16,17,23] nor a prerequisite for rapid transcriptional response [17]. These findings suggest that epigenetic mechanisms have important roles in stem cell identity [24,25], but may also guide differentiation and fate decisions [26,27].

In this light, molecular tools that disrupt global epigenetic mechanisms have the potential to reveal the broader spectrum of genetic circuits operating in stem cells. Among them, the pharmacological agent Trichostatin A (TSA) is particularly potent, inhibiting the enzymatic activity of deacetylases and thus promoting histone acetylation. TSA, by its universal action, provides an entry-point for an overall assessment of the importance of histone modifications on stem cell biology.

To evaluate the importance of histone acetylation on ES cell differentiation, we treated cells with the histone deacetylase inhibitor TSA and examined gene expression changes using Affymetrix gene chips and epigenetic changes using chromatin immunoprecipitation (ChIP) assays. TSA treatment leads to down-regulation of Nanog along with a large group of genes that are characteristic of the undifferentiated state and up-regulation of mesodernal and neuro-ectodermal marker genes. We show here that TSA accelerates the early stages of stem cell differentiation by the global increase of activatory histone modifications and gene-specific changes in the balance between K4 and K27 trimethylations. Both gene expression and epigenetic changes resemble those that appear during embryoid body differentiation.

## Results

### Inhibition of histone deacetylase activity induces phenotypic changes and Nanog suppression in undifferentiated ES cells

**Figure 1:**
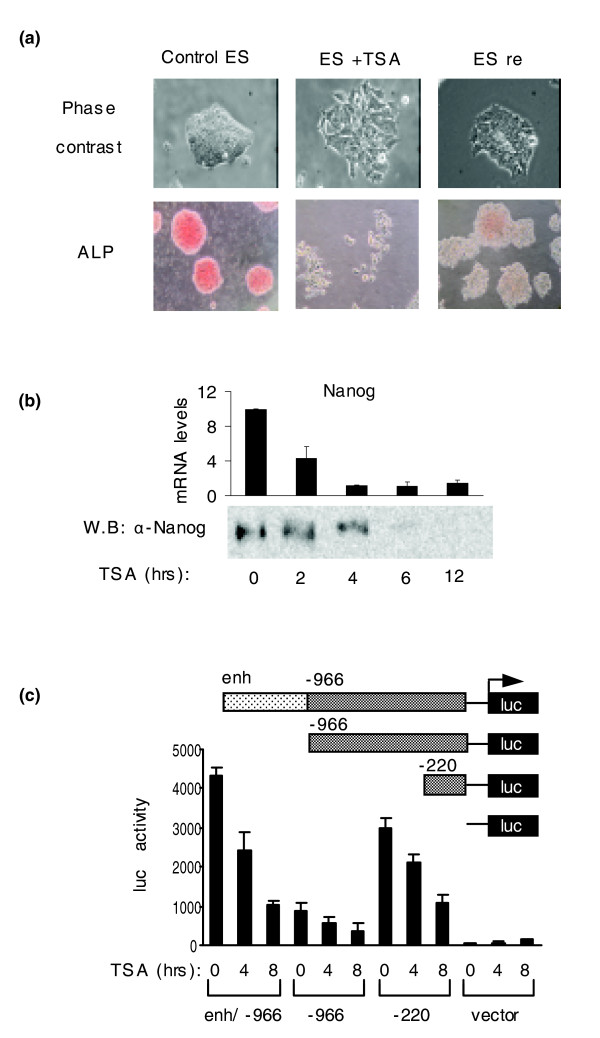
Cell morphology and Nanog expression after TSA treatment. **(a) **ES cells were treated with 50 nM TSA for 12 h and then released from TSA for an additional 12 h. Cell morphology and ALP staining of the three states (ES control, ES+TSA and ES re are shown. **(b) **Nanog mRNA and protein levels after 2, 4, 6 and 12 h of TSA treatment (50 nM). **(c) **Luc activity of *Nanog *promoter/enhancer domains. ES cells were transfected with the indicated fusions of *Nanog *promoter/enhancer fragments to the luciferase reporter gene. Transfected cells were treated with TSA for 0, 4 and 8 h.

To examine the role of histone acetylation on the differentiation state of mouse ES cells, we employed the histone deacetylase inhibitor TSA. We first tested the effect of different TSA concentrations on the mouse CGR8 ES cell line cultivated in the presence of LIF. When 10-50 nM concentrations were used, we observed morphological changes that depended on the concentration and duration of the treatment (not shown). Figure 1a shows a phase contrast morphology and alkaline phosphatase staining (ALP) of mES cells subjected to 50 nM TSA for 12 h. Control cells form round and compact colonies, which stain > 90% positive for ALP. After treatment with 50 nM TSA for 12 h, 70% of the colonies are disrupted and the cells become flattened and negative for ALP; the other 30% show a loose morphology containing a mixed population of ALP-positive and -negative cells.

To identify possible molecular changes underlying the TSA-induced phenotypic transformation, we analyzed the expression of Nanog, one of the master regulators of ES cell identity. Nanog is down-regulated by TSA in a dose-dependent manner, with a concentration of 50 nM having maximal effect (Figure S1A in Additional data file 1). Figure 1b shows that Nanog mRNA levels decline very rapidly starting within the first 2 h of treatment with 50 nM TSA and are minimal by 4 h. Nanog protein levels drop with slower kinetics (Figure 1b). Similar to Nanog, Oct4 and Sox2 mRNA (Figure S2 in Additional data file 1) and protein levels (Figure S1B in Additional data file 1) were reduced during TSA treatment.

In order to test if the rapid Nanog suppression is due to transcriptional silencing, we examined the effect of TSA on the activity of different Nanog promoter fragments cloned in front of the luciferase reporter gene (Figure 1c). Two Nanog promoter fragments extending 966 or 220 bp upstream from the transcriptional start site are both suppressed by TSA (Figure 1c, -966, -220). The proximal promoter has higher activity, possibly because it is deprived of negative regulatory elements that reside within upstream regions. Interestingly, when the Nanog enhancer, located 5 kb upstream of the transcription start site and containing a Nanog auto-regulatory site [28], is fused to the -966 promoter (Figure 1c, enh/-966), it produces a stronger element that is more robustly repressed by TSA than either of the two promoter fragments (Figure 1c). These findings suggest that loss of Nanog expression after TSA treatment is due to transcriptional repression. Moreover, it appears that the effect is mediated by both the proximal promoter, which harbors a composite Oct-4/Sox2 binding site known to regulate Nanog expression [29,30], and the distal enhancer where Nanog binding sites reside [28].

### Microarray analysis of the global TSA effects

The observed morphological effects of TSA and Nanog repression might be indicative of a global and rapid assault on the self-renewal capacity of ES cells, with possibly a simultaneous launch of differentiation. To test this idea, we performed Affymetrix microarray analysis using RNA samples isolated after TSA treatment of ES cells for 6 h to gauge early events and 12 h to test putative secondary effects. The data show extensive changes in the ES transcriptome (Additional data file 2), suggesting that a large fraction of the genome was transcriptionally reprogrammed. Of the differentially expressed genes in TSA-treated compared to control ES cells, 792 genes were down-regulated and 1,376 up-regulated at the 2-fold change cut-off value (Additional data file 2). The gene chip data were validated by real time RT-PCR for 20 selected genes (Figure S3 in Additional data file 1).

A selection of up- and down-regulated genes is presented in Table 1. The category of down-regulated genes contains those encoding important regulators of pluripotency, including Nanog, Sall4, Klf4, Sox2 and Oct4, and other genes typical of the undifferentiated state, such as *Rex1*/*Zfp42*, *FoxD3*, *Gdf3*, *Nr0b1*, *Eras*, *Rif1*, *Tbx3 *and *Esrrb *[2]. It also contains genes encoding a group of chromatin and transcription regulators, such as the histone acetyltransferase PCAF, the H3K9 methyl transferase Suv39, the H3K9 demethylases Jmjd1a and Jmjd2c, the H3K27 demethylase Utx, the Polycomb factors Bmi1, Cbx5, Suz12 and Eed (Table 1) and the transforming growth factor-β/activin signaling pathway members Inhbb, Gdf3 and Lefty2. Among the early and strongly TSA-supressed genes are *Sall1*, *Gli2 *and *Klf2*, which have not been previously associated with regulation of pluripotency and may be novel candidates.

**Table 1:** Functional annotation (biological process) and mRNA fold change of selected TSA (6 and 12 h) down- and up-regulated genes

Gene symbol	Fold change 0 to 6 hrs TSA	Fold change 0 to 12 hrs TSA	F-test_*p*-value	Function (biological process)
*Inhbb*	-11.91	-11.93	2.33E-08	Growth
*Sall1*	-10.95	-16.09	1.06E-05	System development
*Nanog*	- 9.87	-15.24	2.27E-05	Stem cell division
*Gdf3*	- 8.11	-12.56	9.83E-07	Growth
*Lefty2*	- 5.7	-16.58	6.87E-06	Development
*Gli2*	- 5.61	-8.38	4.34E-08	Regulation of transcription, DNA-dependent
*Klf2*	- 4.73	-10.39	4.48E-08	Positive regulation of transcription
*Zfp42/Rex*	- 4.62	-5.00	1.01E-05	Regulation of transcription, DNA-dependent
*Foxd3*	- 4.59	-4.66	3.74E-05	Regulation of transcription, DNA-dependent
*Tbx3*	- 4.46	-6.10	9.29E-05	Development, cell aging, negative regulation of transcription
*Eras*	-4.22	-5.94	3.11E-06	Small GTPase mediated signal transduction
*Nr0b1*	-4.11	-13.39	1.70E-06	Negative regulation of transcription
*Rif1*	-4.03	-3.82	1.54E-04	Response to stress, chromosome maintenance
*Lefty1*	-3.66	-9.11	1.13E-06	Development, cell growth
*Suv39h1*	-3.50	-4.87	2.38E-06	Chromatin assembly or disassembly, chromatin modification
*Sall4*	-3.47	-3.34	2.47E-03	Stem cell pluripotency
*Utx*	-3.61	-2.81	1.68E-04	Chromatin modification
*Pcaf*	-3.29	-5.15	3.79E-05	Regulation of transcription, DNA-dependent
*Bmi1*	-3.39	-1.80	2.39E-06	Chromatin modification, somatic stem cell division, development
*Klf4*	-3.02	-4.09	1.59E-06	Regulation of transcription, DNA-dependent
*Fgf4*	-2.95	-3.47	2.23E-06	Stem cell maintenance, regulation of cell cycle
*Zic3*	-2.87	-3.28	2.38E-05	Regulation of transcription
*Esrrb*	-2.64	-4.26	2.96E-04	Regulation of transcription, DNA-dependent
*Sox2*	-2.43	-1.63	2.03E-04	Cell fate specification, regulation of transcription, DNA-dependent
*Jmjd1a*	-2.20	-2.94	8.14E-08	Chromatin modification
*Suz12*	-2.10	-1.87	1.58E-03	Chromatin modification
*Jmjd2c*	-2.20	-2.27	2.28E-04	Chromatin modification
*Tcl1*	-1.85	-3.13	1.09E-04	Regulation of transcription
*Eed*	-1.80	-2.05	2.37E-05	Imprinting
*Cbx5*	-1.42	-2.21	1.76E-03	Chromatin assembly or disassembly
*Pou5f1*	-1.09	-1.74	6.04E-04	Stem cell maintenance
*Egr1*	28.37	40.75	1.53E-06	Regulation of transcription, DNA-dependent
*H1f0*	13.39	22.33	4.45E-06	Nucleosome assembly
*Hist1h1c*	12.34	17.88	1.01E-03	Nucleosome assembly, chromosome organization and biogenesis
*Pdgfrb*	9.78	10.77	3.98E-06	Protein tyrosine kinase signaling pathway
*Fos*	8.02	11.35	9.00E-07	Regulation of cell cycle, regulation of transcription, neurogenesis
*Ndrg4*	7.87	8.15	9.85E-06	Cell differentiation, development
*Hist3h2a*	5.98	11.17	7.97E-04	Nucleosome, chromosome
*Creg1*	4.65	5.32	2.84E-06	Regulation of transcription, DNA-dependent
*Ttll1*	4.47	5.13	7.84E-06	Protein modification
*Nnat*	4.05	8.37	1.04E-03	Development
*Hoxa1*	4.05	5.34	8.61E-06	Anterior/posterior pattern formation, hindbrain development
*Edg3*	3.88	4.82	1.53E-04	G-protein signaling, positive regulation of cell proliferation
*Cacna1b*	3.83	7.21	8.76E-04	Neurotransmitter secretion, regulation of heart contraction
*Idb2*	3.49	7.12	3.42E-04	Development, heart development, lymph gland development
*Hoxb13*	3.33	4.45	6.74E-06	Pattern specification, organogenesis, regulation of growth
*Cacna1h*	3.15	5.50	1.45E-05	Calcium ion transport
*Cbx4*	3.14	4.85	5.13E-05	Chromatin assembly or disassembly, chromatin modification
*Sirt7*	2.69	2.68	1.05E-04	Chromatin silencing, regulation of transcription, DNA-dependent
*Mlf1*	2.29	12.44	6.88E-05	Cell differentiation
*Ctgf*	2.12	9.88	6.80E-04	Ossification, angiogenesis, regulation of cell growth, cell adhesion
*Cbx2*	1.81	2.00	6.16E-05	Chromatin assembly or disassembly, chromatin modification
*Wif1*	1.41	6.33	2.38E-06	Negative regulation of Wnt receptor signaling pathway

In the category of up-regulated transcripts, we detected: genes of the neural lineage, such as *Hoxa1*, *Hoxb13*, *Nnat*, and *Mbp*; genes of the hematopoietic lineage, for example, *Mlf1*; vascular and neuronal differentiation related genes like *Pdgfrβ*; a group of genes encoding histones, including the differentiation-specific histone H1f0; and genes encoding the connective tissue growth factor Ctgf and the endothelial-specific receptor Edg3. In addition, TSA activates the immediate-early response genes *Egr1*, *Fos *and *JunB*, which have been associated with cell proliferation, differentiation, transformation and apoptosis.

**Figure 2:**
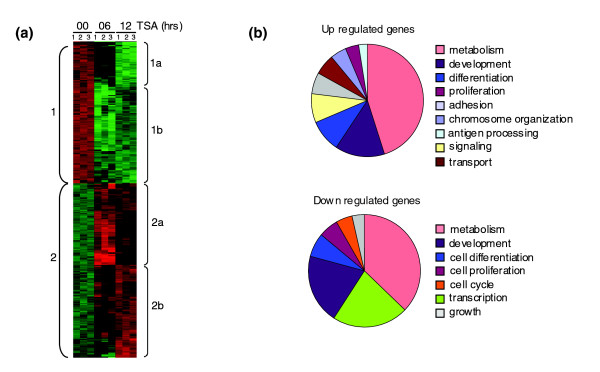
Gene expression changes after TSA treatment and functional annotation of affected genes. **(a) **Hierarchical cluster analysis of the TSA-induced transcriptome. The numbers 1, 2, and 3 at the top represent three biological replicates of the experiment. Brackets on the left mark the two major clusters of down-regulates (cluster 1) and up-regulated (cluster 2) genes. Brackets on the right mark the subclusters of the four different expression profiles observed, that is, down-regulation after 12 h of TSA treatment (subcluster 1a) or after 6 h (subcluster 1b), and up-regulation after 6 h (subcluster 2a) or 12 h (subcluster 2b). **(b) **Pie charts representing the functional annotation of up- or down-regulated genes. Transcripts differentially expressed by ≥ 4-fold after 6 or 12 h of TSA treatment were used for all the above experiments.

To narrow down the genes under study, we focused on the most significant changes and chose to analyze genes with expression changes equal to or greater than four-fold. Using this gene list we performed a hierarchical clustering in order to uncover genes that respond similarly to TSA treatment, pointing to a possible functional interconnection (Figure 2a; Figure S4 in Additional data file 1). This analysis showed the existence of two major clusters of down-regulated (cluster 1) and up-regulated (cluster 2) genes and unveiled a further division of each cluster into two subclusters (Additional data file 3). Subclusters 1b (117 transcripts) and 2a (111 transcripts) show major changes at 6 h whereas subclusters 1a (60 transcripts) and 2b (112 transcripts) do so at 12 h. To functionally categorize the gene clusters and subclusters, we used the Database for Annotation, Visualization, and Integrated Discovery (DAVID) [31] to obtain Gene Ontology annotations for the category of 'biological process' (Table 2). Down-regulated transcripts (subclusters 1a and 1b) contain genes that fall in the categories of metabolism (*Cbr3*, *Tdh*, *Enpp3*, *Cacna1a*, *Cul1*), development/morphogenesis (*Nanog*, *Nr0b1*, *Sall1*, *Gli2*, *Lefty1*, *Lefty2*) and growth (*Gdf3*, *Gja1*, *Socs2*, *Inhbb*). In addition, genes from subcluster 1b fall in the category of transcription (*Tcfap4*, *Gtf2I*, *Ubtf*, *Suv39h1*). Up-regulated genes from the early-induced subcluster 2a participate in neural system development (*Sema4f*, *Hoxa1*, *Stxbp1*), whereas subcluster 2b (induced after 12 h) contains genes that take part in angiogenesis and hemopoiesis. Additionally, subcluster 2a members participate in cell organization/biogenesis (*CenpJ*, *Tubb2a*, *Sept4*) and intracellular signaling (*Edg3*, *Rnd1*, *Mknk2*), while subcluster 2b members have been implicated in metabolism (*Hsdl2*, *Tgm2*, *Pygl*), chromosome organization, that is, nucleosome and chromatin assembly/disassembly, (*H1h2bf*, *H1h2bc*, *H12bp*, *H2h2be*, *H1fx*) and antigen processing (*H2-T3*, *H2-K1*, *CD74*).

**Table 2:** Major functional categories of the four subclusters from the hierarchical clustering (Figure 2b) and the respective genes

Subcluster	Genes	Function
1a	*Lefty1*, *Lefty2*, *Nr0b1*, *Mcf2*, *Notch4*,	Metabolism
	*Foxh1*, *Ubtf*, *Gtf2i*, *Tdh*, *Upp1*, *Fkbp9*,	Development: embryonic, tube
	*Enpp3*, *Socs2*, *Cacna1a*, *Pycard*, *Enah*,	Morphogenesis
	*Lef1*, *Esrrb*, *Rnf125*, *Ddc*, *Bcat1*, *Dpp4*,	Pattern specification
	*Ptpmt1*, *Ctbp2*, *Pml*, *Ptbp2*, *Hnrpa2b1*	Cell differentiation
		Growth
1b	*Nanog*, *Tbx3*, *Sall1*, *Gdf3*, *Klf3*, *Klf4*,	Metabolism
	*Zic3*, *Zic5*, *FoxD3*, *Gli2*, *Gja1*, *Otx2*,	Transcription
	*Zbtb7a*, *Nsd1*, *Arid1a*, *NFIb*, *Pitx2*, *Rarg*, *Suv39H1*, *Cul1*, *Ppap2b*, *Pdgfc*, *Tns3*,	Development: embryonic, organ, tube
	*Inhbb*, *Fn1*, *Tcfap4*, *Nr5a2*, *MllT6*, *Irf2bp2*, *Pcaf*, *Zfp42*, *Zmynd11*, *Ncor1*,	Morphogenesis: embryonic, organ, tube
	*MllT10*, *Tgif*, *Trps1*, *Emp1*, *Pkd2*, *Spry2*,	Cell proliferation
	*Gjb3*, *Fgf4*, *Vegfc*, *Hsd17B11*, *Cbr3*,	Growth
	*Fbxo15*, *Dusp27*, *Frrs1*, *Cdk6*, *Epb4.9*, *Irak3*, *Spry4*, *Manba*, *Folr1*	
2a	*Sema4f*, *Mbp*, *Cnp*, *Stxbp1*, *Hoxa1*, *Fos*, *Farp2*, *Sept4*, *Krt1-18*, *H1f0*, *Tubb2a*,	Cell organization and biogenesis
	*Dnajc12*, *Cenpj*, *Spire1*, *Pappa2*, *Sh2b2*, *Tax1bp3*, *Rnd1*, *Arhgap29*, *Edg3*, *Mknk2*, *Errf1*, *Rap40b*, *Pdgfrb*	System development: nervous system development; neurogenesis; cell development
		Intracellular signaling
2b	*Id2*, *Id4*, *Wif1*, *Hoxb13*, *Prkar1b*, *Prkar2b*,	Metabolism
	*Zfp36*, *Cd74*, *Thy1*, *Serpine1*, *Dhcr7*, *Pdlim7*, *Ctgf*, *Mlf1*, *Lgals1*, *Kif3a*, *Irf8*, *Hsdl2*, *H1h2bf*, *H1h2bp*, *H1h2bc*, *H2h3c1*, *Junb*, *Cbx4*, *Hspa1a*, *Hspa1b*, *Hspa2*, *Acaa1b*, *H1fx*, *Psmb9*, *H2-T3*, *H2-K1*, *Hist1h2bn*	Development: organ development/morphogenesis; vasculature development; angiogenesis; hemopoiesis/blood vessel development
		Cell differentiation
		Chromosome organization
		Immune response

An overview of the various gene function categories is shown as pie charts for clusters 1 and 2 in Figure 2b. Comparison of the pie chart representations between up- and down-regulated genes reveals some interesting differences. For example, regulators of cell cycle, cell growth and transcription are represented in the cluster of down-regulated genes, suggesting a strong effect of TSA treatment on the self-renewal machinery. On the other hand, signaling and adhesion molecules become evident in the pie chart of induced genes, indicating the appearance of new response mechanisms to environmental cues. A full list of genes from each subcluster and the corresponding biological process annotations are included in Additional data files 3-7.

### Histone deacetylase inhibition effects resemble gene expression changes appearing during embryoid body differentiation

**Figure 3:**
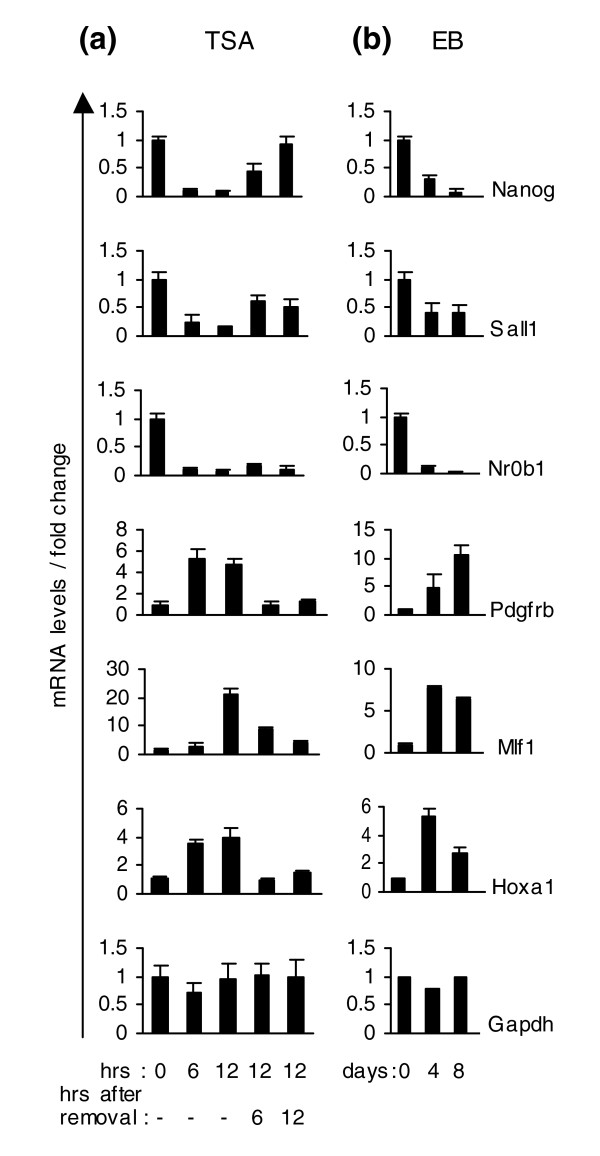
Expression patterns of selected genes in TSA-treated ES cells and EBs. mRNA levels of *Nanog*, *Sall1*, *Nr0b1*, *Pdgfrb1*, *Mlf1 and Hoxa1 *in: **(a) **ES cells treated with 50 nM TSA for 6 and 12 h. After 12 h of treatment, TSA was removed and cells were cultivated for an additional period of 6 and 12 h. **(b) **EBs 0, 4 and 8 days old. Control mRNA levels (0 h TSA/0 days EBs) were set to 1 and normalized with glyceraldehyde phosphate dehydrogenase. mRNA levels were analyzed with real-time PCR.

To examine how gene expression modulation caused by TSA corresponds to changes taking place during the 'natural' *in vitro *differentiation process, we placed ES cells in hanging drops to form embryoid bodies (EBs) and allowed them to differentiate without LIF. We then analyzed the mRNA levels of six genes from Table 1 that are strongly affected, negatively or positively, by TSA. These genes were those encoding Nanog, the spalt homologue Sall1, the orphan nuclear receptor Nr0b1, the vascular and neuronal differentiation related receptor Pdgfrβ, and the hematopoietic lineage switch gene *Mlf1 *and the homeotic *Hoxa1 *gene. Figure 3a shows that TSA causes a very rapid repression of Nanog, Nr0b1 and Sall1, a rapid but more gradual induction of Pdgfrβ and Hoxa1, and a late increase of Mlf1 mRNA levels.

During embryoid body differentiation, suppression or induction of individual genes takes place in distinct time frames. To compare the TSA effect with EB differentiation, days 4 and 8 were chosen as the most indicative even though gene expression changes were observed earlier (EBs days 2 and 3). Nanog, Nr0b1 and Sall1 were also found to be repressed during EB formation whereas Pdgfrβ, Mlf1 and Hoxa1 genes were activated. (Figure 3b). Therefore, the expression of these genes in embryoid bodies is regulated in the same direction as after TSA treatment, albeit at much slower paces (Figure 3b). This was true for the majority of genes checked so far.

### The effect of TSA is partially reversible

We next asked if the differentiation imposed by TSA is reversible. To answer this question, ES cells were treated with TSA for 12 h and then cultured for an additional 6 and 12 h without TSA (ES re). We observed that the morphological changes induced by TSA were gradually reversed with the emergence of compact colonies (approximately 70% of the control), which are indicative of the undifferentiated state (Figure 1a, ES re). These colonies stain weakly for ALP in their center (Figure 1a). RT-PCR analysis demonstrated that the expression of Nanog, Mlf1, Hoxa1 and Pdgfrβ was fully reversed to pre-treatment, undifferentiated ES cell levels (Figure 3a). In contrast, the mRNA levels of Nr0b1 and Sall1 did not recover fully. In agreement with the reduction of Nanog mRNA, FACS analysis showed that the population of cells expressing Nanog above control antibody levels was reduced by TSA and then recovered upon release from TSA treatment (Figure S5 in Additional data file 1). Further analysis of three well characterized pluripotency factors, Oct4, Sox2 and Zfp42/Rex1, revealed that the mRNA levels of Oct4 and Sox2 but not of Zfp42/Rex1 were restored after TSA removal (Figure S2 in Additional data file 1). In conclusion, although the morphological changes that are caused by TSA treatment are largely reversed, expression of individual genes can undergo either fully or partially reversible alterations. The partial return to the ES cell phenotype suggests that histone deacetylase inhibition alone does not appear to fully commit ES cells to differentiation.

**Figure 4:**
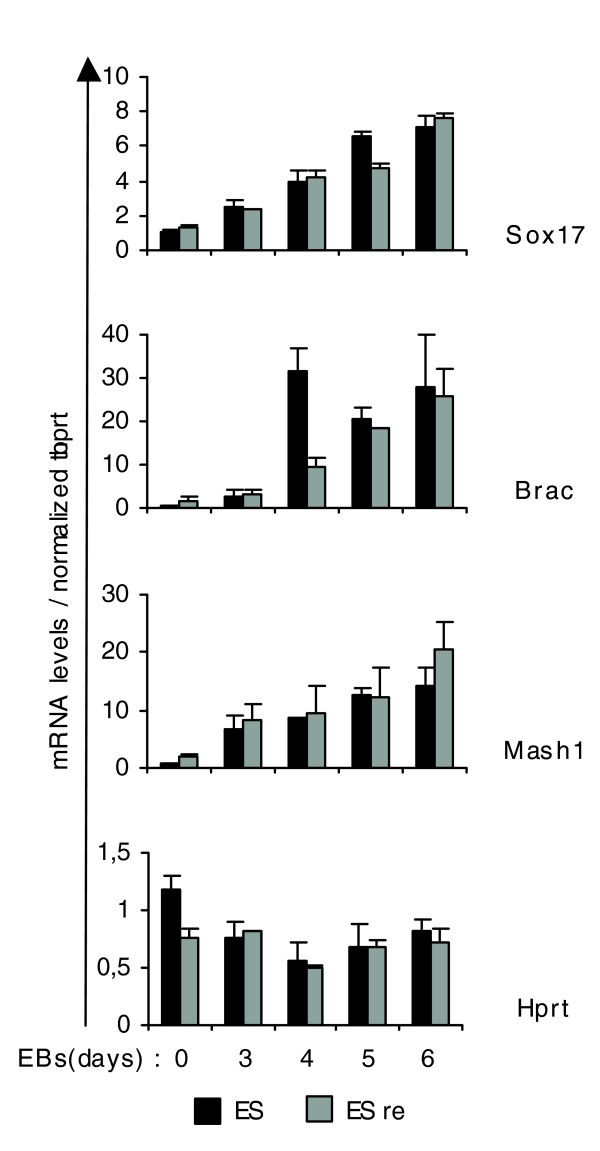
mRNA levels of *Sox17*, *Brac and Mash1 *during EB formation of control and released ES cells (ES re). Control and released ES cells grown at clonal density were placed in hanging drops to form EBs. mRNA levels of the indicated genes were measured using real time RT-PCR analysis and were normalized to Hprt.

In order to examine the competence of the 'ES re' cells to contribute to the three germ layers, we placed them in hanging drops and observed that they formed EBs of normal morphology. We then checked for expression of markers of the three germ layers, endoderm (Sox17), mesoderm (Brachyury) and ectoderm (Mash1). As shown in Figure 4, Sox 17 and Mash1 followed the same expression patterns as in wild-type cells whereas Brachyury was up-regulated one day later than in wild-type cells. These results show that 'ES re' cells are still capable of acquiring either one of the three cell fates.

### Histone modification changes correlate with gene expression reprogramming

**Figure 5:**
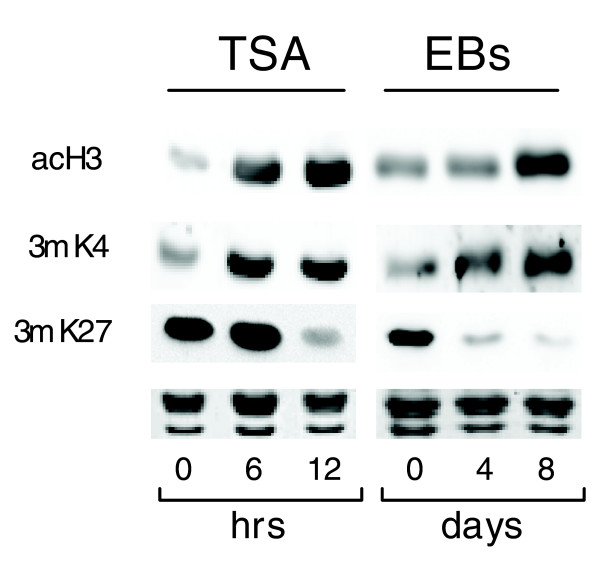
Analysis of bulk histone modifications in TSA-treated ES cells and EBs. Global levels of histone H3 acetylation (acH3), and lysine 4 (3mK4) and lysine 27 (3mK27) trimethylation, employing immunoblotting with specific antibodies. Equal loading was controlled with Coomassie blue staining.

To gain insight into the molecular mechanisms whereby TSA triggers stem cell differentiation, we analyzed the dynamics of histone H3 modifications after TSA treatment and compared them to changes taking place during EB formation. In both cases, we observed an increase in the global amounts of two activatory modifications, H3 acetylation and K4 trimethylation and a decrease in repressive K27 trimethylation (Figure 5). These results show that TSA treatment instigates a global enhancement of activation-linked epigenetic marks that also appears during the natural ES cell differentiation process.

**Figure 6:**
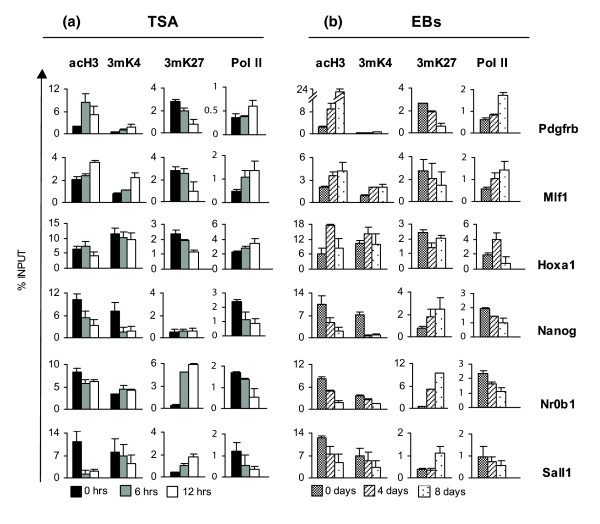
Histone modification changes and RNA polymerase (Pol) II levels on gene promoters during TSA treatment and EB formation. Histone modifications (H3 acetylation (acH3), and lysine 4 (3mK4) and lysine 27 (3mK27) trimethylation) and Pol II levels on the promoters of **(a) **activated genes (*Pdgfrβ*, *Mlf1*, *Hoxa1*) and **(b) **repressed genes (*Nanog*, *Nr0b1*, *Sall1*) during TSA treatment (left) or EB differentiation (right). Modification levels were estimated using ChIP assays. Results are expressed as percent of the input chromatin.

However, analysis of individual genes using ChIP assays uncovered a complex, gene-specific pattern of histone modifications (Figure 5). In this experiment, we first examined the histone modifications on the promoters of three genes that were up-regulated by TSA, namely *Pdgfrβ*, *Mlf1 *and *Hoxa1 *(Figure 3a). During activation of *Pdgfrβ *and *Mlf1 *by TSA, we detected an increase in H3 acetylation and H3K4 trimethylation and a decrease in H3K27 trimethylation (Figure 6a). Analysis during EB formation (Figure 6b) gave similar results. In contrast, we found that the neural lineage gene *Hoxa1 *had significant concurrent activatory (K4) and suppressive (K27) methylations in the undifferentiated state (in agreement with the bivalent structure model [20]). Upon activation by TSA, we observed a reduction in K27 trimethylation levels and maintenance of K4 trimethylation and H3 acetylation levels. Thus, activation of *Hoxa1 *expression after TSA treatment relies on loss of suppressive modifications. *Hoxa1 *expression during EB differentiation is correlated with a transient increase in H3 acetylation, matching the *Hoxa1 *maximal activation (Figure 3a), and a transient decrease in K27 trimethylation (Figure 6b). In contrast to this finding, another study [32] has reported that TSA-induced activation of *Hoxa1 *is not correlated with a drop in K27 trimethylation. However, in that case, ChIP analysis was performed on a retinoic acid-regulated enhancer at the 3' end of the gene as opposed to our analysis, which is based on the 5' promoter region, proximal to the transcriptional start site.

ChIP analysis was also performed for genes down-regulated by TSA treatment. Along with *Nanog *mRNA repression, we detected a gradual decrease of H3 acetylation and an abrupt drop in H3K4 trimethylation of its promoter without an increase in the repressive H3K27 trimethylation (Figure 6a). During EB differentiation, Nanog inactivation similarly correlates with a decrease in both H3 acetylation and H3K4 trimethylation, but in this case gene suppression is also accompanied by an increase in H3K27 trimethylation (Figure 6b). Unlike *Nanog*, repression of *Nr0b1 *was accompanied by a robust increase in H3K27 trimethylation, and no significant decrease of either acetylation or H3K4 trimethylation (Figure 6a). In EBs, H3K27 trimethylation was also increased but both activatory modifications were reduced. Finally, the *Sall1 *promoter represented an intermediate situation of repression, connected to a strong decrease in acetylation, a decrease in H3K4 trimethylation and a rise in H3K27 trimethylation (Figure 6a). Similar changes accompany *Sall1 *deactivation during EB differentiation.

**Figure 7:**
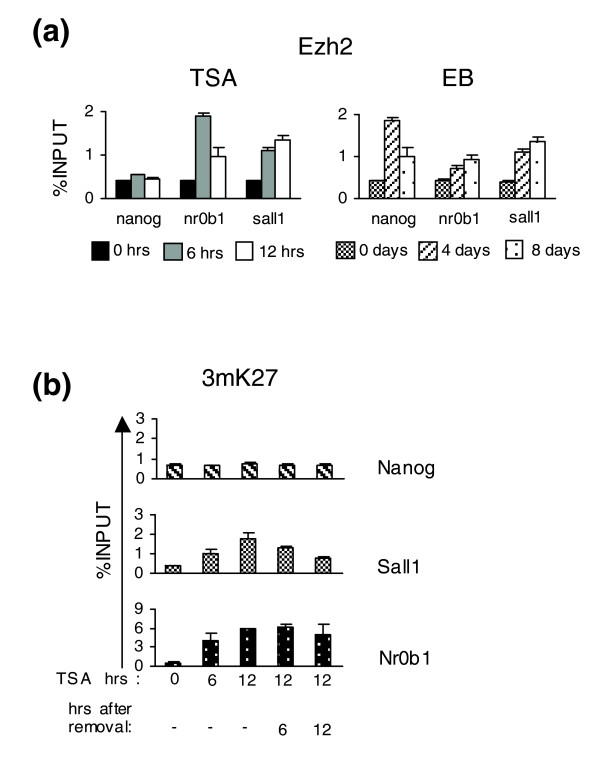
Ezh2 and H3K27 trimethylation levels on the promoters of *Nanog*, *Sall1 and Nr0b1*. **(a) **ChIP assays using an anti-Ezh2 antibody on the promoters of *Nanog*, *Sall1 and Nr0b1 *during TSA treatment (6 and 12 h) and EB formation (4 and 8 days). **(b) **ChIP assays using an anti-3mK27 antibody were performed for the promoters of TSA down-regulated genes *Nanog*, *Sall1 and Nr0b1 *after 6 and 12 h of TSA treatment and further cultivation without TSA for an additional 6 and 12 h.

Trimethylation of K27 is catalyzed by Enhancer of Zeste 2 (Ezh2), a methyl-transferase component of the PRC2 complex. Employing ChIP assays, we confirmed the recruitment of Ezh2 on *Nanog*, *Sall1 *and *Nr0b1 *promoters (Figure 7a), in agreement with the appearance of K27 trimethylation during suppression either by TSA or in EBs (Figure 6a,b). Gene activation or repression of all six genes in both TSA-treated ES cells and EBs correlates with a respective increase or decrease in promoter-bound RNA polymerase II (Figure 6a,b). These results indicate that the observed chromatin modifications correlate well with the expected recruitment of transcriptional regulators and enzymes to the corresponding gene promoters.

Correlating ChIP data with changes in mRNA levels, it appears that H3K27 trimethylation might predispose individual genes for stable repression. For example, *Sall1 *and *Nr0b1*, which show an increase in H3K27 trimethylation following addition of TSA (Figure 6a), do not regain full expression after release from TSA (Figure 3a). Nanog, on the other hand, which is not marked by H3K27 trimethylation when repressed, regains full expression following TSA release. To further strengthen this idea, we prepared chromatin samples from cells treated with TSA for 12 h and then released for 6 and 12 h. ChIP analysis shows that H3K27 trimethylation induced by TSA is maintained on the *Nr0b1 *promoter and, partially, on the *Sall1 *promoter even after TSA removal (Figure 7b), in agreement with the irreversible repression of the two genes (Figure 3a). Collectively, by analyzing six different genes during TSA treatment and EB formation, we have encountered similar but gene-specific combinations of promoter chromatin modifications that correlate with expression state.

## Discussion

ES cells can differentiate along various pathways and this process is linked to their unusually open chromatin structure [24]. In this report, we have undertaken a transcriptomic approach in order to analyze how histone deacetylase inhibition affects the self-renewal activity or differentiation of mouse ES cells. In parallel, we have examined histone modification changes that correlate with transcriptional reprogramming.

Gene expression profiling following histone deacetylase inhibition in ES cells revealed two major gene clusters: genes highly expressed in undifferentiated cells that are suppressed by TSA and genes not expressed in ES cells that are activated by TSA. Expression levels of these genes change in an opposite way. This may reflect a cross-regulation between genes of the two clusters, or the existence of common regulators that modulate the simultaneous repression or induction of selective targets. The second possibility seems valid in the light of the discovery that binding of Nanog/Oct4/Sox2 complexes to promoter regions maintains different gene targets in either active or inactive states [6].

*Nanog*, among other genes encoding pluripotency factors, is an early and strongly (approximately 15-fold) TSA-suppressed gene. Studying the activity of different *Nanog *promoter/enhancer regions, we found that this effect is transcriptional and depends on the proximal promoter (-220 kb) and the distal enhancer (-5 kb). Oct4 and Sox2 are known regulators of the *Nanog *proximal promoter. Since the genes for Oct4 and Sox2 are only weakly repressed, it is unlikely that they are the main mediators of the TSA effect on the *Nanog *promoter. A fine, site-specific, mutational analysis could clarify the regulatory elements targeted by TSA, as well as the cognate DNA binding factors.

Our microarray analysis indicates that histone deacetylase inhibition induces the exit from the undifferentiated state as revealed by the suppression of most known pluripotency factors (Tables 1 and 2). Therefore, we propose that many other genes in this category, that is, those suppressed by TSA, might also be involved in the regulation of self-renewal. Despite the swift loss of many pluripotency factors, induction of differentiation was not directed to a particular lineage. Instead, we detected up-regulation of various genes associated with neural and mesodermal, but not endodermal, differentiation, indicating that TSA leads cells to an intermediate stage between the undifferentiated and the finally differentiated states.

Histone deacetylase inhibition induces gene expression changes and chromatin modifications that also take place during the 'natural' differentiation process, albeit in a compressed time frame. For example, changes appearing within 12 hours of TSA treatment manifest during an eight-day period of EB differentiation. On a global scale, the TSA effects include increases in the activating modifications histone H3 acetylation and K4 trimethylation and a concomitant loss of the repressive K27 trimethylation. As previous studies have shown that undifferentiated ES cells bear increased activation marks when compared to their differentiated descendants [15], our results suggest that before full commitment to a differentiated phenotype, there might be a window of chromatin 'over-permissiveness' characterized by an increase in activation marks (Figure 5). TSA might facilitate this transient phase, thus accelerating cell differentiation, possibly by reorganizing the chromatin structure [33].

TSA can have different effects depending on the dose and duration of the treatment and the target cell differentiation state. Brief treatment with 300 nM TSA provoked changes in the allelic chromatin conformation of the imprinted U2af1-rs1 locus in mouse ES cells but not fibroblasts [34]. In another report [35], the addition of TSA during EB formation resulted in inhibition of differentiation, reflected in persistent ALP staining. It seems, therefore, that when ES cells are disaggregated and put in a specific differentiation milieu, they can not differentiate in the presence of TSA. In line with our results, TSA was shown to promote myocardial differentiation when added to seven-day-old EBs [36] and neuronal differentiation when added to embryonic neural stem cells [37].

Histone deacetylase inhibition is generally considered an inducer of gene activation by increasing H3 acetylation levels. However, examination of the histone modification changes that occur on the promoters of six genes targeted by TSA has revealed complex, gene-specific regulation patterns. Even though TSA inhibits both type I and II, histone deacetylases (HDACs) it can induce a decrease in histone H3 acetylation on the promoters of some (Figure 6), but not all, suppressed genes, as suggested in a previous report [38]. Transcriptional repression induced by TSA might be an indirect effect connected to reduction of PCAF acetyltransferase levels or an increase in Sirt7 deacetylase mRNA levels (Table 1; Additional data file 2). In this scenario, we would have expected suppressed genes to follow slower kinetics compared to activated ones. Instead, genes that are strongly suppressed by TSA, such as *Nanog*, *Nr0b1 *and *Sall1*, follow very rapid kinetics (Figure S6 in Additional data file 1), arguing for a direct rather than an indirect effect. Moreover, even low doses of TSA can repress *Nanog *(Figure S1A in Additional data file 1).

The pleiotropy of the TSA effects is obvious when considering the regulation of *Nanog *by HDAC1. HDAC1 levels on the *Nanog *promoter increased after TSA treatment (Figure S7 in Additional data file 1), and even though HDAC1 activity is inhibited by TSA, Nanog chromatin is still deacetylated (Figure 6a). In addition, by comparing our data to a microarray analysis of HDAC1 knock out ES cells [39], we found no significant overlap between the two experiments, pointing to the multiplicity of the mammalian deacetylases [38] and their potential non-histone target proteins. We can not exclude the possibility that the acetylation state of non-histone proteins may account for the observed actions of TSA. To explore this possibility, it would be important to examine the acetylation of factors important for the ES differentiation state.

Our study shows that besides acetylation, histone deacetylase inhibition in ES cells dramatically affects methylation of lysines 4 and 27 of histone H3. We have documented for the first time a rapid decrease in K4 and K27 trimethylation levels. Until recently, it was difficult to explain such phenomena because methylation was considered a stable mark. Based on the recent discovery of novel enzymes that remove trimethyl-K4 (3mK4) [40,41] and trimethyl-K27 (3mK27) [42,43], we postulate that TSA may modify the recruitment and/or activity of H3 methylation/demethylation complexes. At the moment, it is not clear how this recruitment on specific promoters might be regulated, leaving unclear how genes are selected for repression or activation. Genome-wide analyses have identified target genes for K4 and K27 trimethylations in ES cells [16,17,22]. However, apart from the case of Hox genes, the DNA target sites that recruit the mammalian Trithorax and Polycomb complexes remain largely unknown.

We show that the establishment of H3K27 trimethylation correlates well with stable transcriptional reprogramming. For example, Nanog repression, which is reversible in TSA-treated ES cells, is not accompanied by an increase in K27 trimethylation, whereas the opposite occurs in EBs, where the gene is permanently silenced. On the other hand, *Nr0b1 *and *Sall1*, which are irreversibly repressed by TSA, show increased K27 trimethylation that is maintained even after removal of TSA (Figure 7b). Thus, restoration of Nanog expression may be the reason for the partial reversal of the cells to the undifferentiated state after TSA removal. With regard to this, genome-wide analysis of H3K27 trimethylation maps in correlation with mRNA levels after TSA removal would be very informative. Collectively, histone deacetylase inhibition is able to disrupt stem cell pluripotency and facilitate early differentiation events, although it is not sufficient for commitment to a differentiation fate.

## Conclusion

This work documents comprehensive gene expression changes that are induced by histone deacetylase inhibition in mouse ES cells. Pluripotency regulators, including Nanog, are repressed while differentiation-associated genes related to the neuro-ectodermal and mesodermal lineages are activated. This transcriptional reprogramming is regulated by changes in histone H3 methylation of lysines 4 and 27. Histone deacetylase inhibitors may have applications in stem cell differentiation and in therapies against tumors that express stemness factors.

## Materials and methods

### Cell culture, antibodies and chemical reagents

CGR8 ES cells were cultivated in GMEM (10% fetal bovine serum, 1,000 units LIF (ESGRO-Chemicon Temecula, CA, USA)). Antibodies were from Upstate (Upstate/Millipore, Billerica, MA, USA) (AcH3, 3mK4H3, 3mK27H3, Ezh2), Chemicon (Nanog) and Santa Cruz (Santa Cruz, CA, USA) (His, RNAP II). TSA was from Sigma (Sigma, Saint Louis, MO, USA).

### Alkaline phosphatase staining

CGR8 cells were fixed with 100% methanol and stained with a solution of 1 mg/ml Fast Red TR salt TM (Sigma) and 200 μg/ml Napthol AS-MX phosphate (Sigma) in 0.1 M Tris pH 9.2.

### Plasmids and transfections

*Nanog *promoter/enhancer fragments were cloned upstream of the luciferase reporter gene in the pGL3-basic vector (Promega Madison, WI, USA). A -966/+50 fragment was obtained by PCR and was cloned in the pGL3 vector. Primers used were: forward, 5'-AGCACAAGGACTGATCGG-3', reverse, 5'-GCAGCCTTCCCACAGAAAG-3'. The enhancer fragment was obtained by PCR and was cloned in front of the -966 fragment in the pGL3 vector. Primers used were: forward, 5'-ATATAGGTACCCCCCTCCCCCACCTGTCCC-3', reverse, 5'-TATATGCTAGCG GCCACATAGCCTTAAGT-3'. For -220/+50 construction, -966/+50 was digested with *Hin*dIII and the excised fragment was cloned in the pGL3 vector. CGR8 cells were transfected using Lipofectamine 2000 (Invitrogen, Carlsbad, CA, USA). The full length *Nanog *cDNA plasmid was kindly provided by P Savatier.

### Chromatin immunoprecipitation assays

ES cells and EBs were fixed with 1% formaldehyde for 10 minutes at room temperature and the reaction was quenched by adding glycine to a final concentration of 0.125 M. ES cells were washed once with ice-cold phosphate-buffered saline (PBS), harvested and washed two more times. EBs were also washed three times with ice-cold PBS. ES cells and EBs were resuspended in lysis buffer (1% SDS, 10 mM EDTA, 50 mM Tris-HCl pH 8.0, 1 mM phenylmethylsulphonyl fluoride (PMSF), 1 ml/10^6 ^cells) and incubated on ice for 10 minutes. The suspension was sonicated 5 times for 1 minute each and 10 μl samples were analyzed by gel electrophoresis (1.5% agarose). Properly sonicated samples were centrifuged at 14,000 rpm, 4°C for 15 minutes and the supernatant was stored at -80°C. We kept 10 μl from each sample as input and 50 μl were immunoprecipitated with 5 μg of relevant antibodies in RIPA buffer (1% Triton X-100, 0.1% deoxycholate (DOC), 140 mM NaCl, 1 mM PMSF) overnight at 4°C under rotation. Protein G beads were incubated in the same conditions with 100 μg/ml sonicated salmon sperm DNA and 1 μg/ml bovine serum albumin in RIPA buffer. Blocked beads and immunoprecipitated samples were combined next day and were incubated under rotation for 3 h at 4°C. The immunoprecipitates were then washed 7 times with RIPA wash buffer (1% Triton-X, 0.1% DOC, 0.1% SDS, 500 mM NaCl, 1 mM PMSF). Input samples and beads were resuspended in 100 μl of 10 mM Tris-HCl pH 8.0, 1 mM EDTA (TE) buffer supplemented with 0.5% SDS and proteinase K to a final concentration of 200 μg/ml and incubated for 3 h at 55°C and overnight at 65°C. The next day samples were phenol-chloroform extracted and ethanol immunoprecipitated with NaOAc and 20 mg of glycogen as a carrier. DNAs from input and immunoprecipitate pellets were resuspended in 50 μl and 250 μl of TE buffer, respectively. The DNA content was analyzed using real-time PCR (5 μl/20 μl reaction). The primers used were: *Nanog *promoter sense, 5'-CTTACTAAGTAGCCCAGTC-3'; *Nanog *promoter antisense, 5'-GTTTATACACGGTTCTTT-3'; *Nr0b1 *promoter sense, 5'-AGTTGGAACAGAGCCCTAAC-3'; *Nr0b1 *promoter antisense, 5'-GCCTTTGGTTGAATGTG-3'; *Sall1 *promoter sense, 5'-TGCGACATGGGTCCTGAG-3'; *Sall1 *promoter antisense, 5'-AATTCTGGAGCGCCTTTGAGT-3'; *Hoxa1 *promoter sense, 5'-GAGCGCGCGTCACCTACAC-3'; *Hoxa1 *promoter antisense, 5'-CTGAGCCGCCTGCGAAAGTT-3'; *Pdgfrb *promoter sense, 5'-GCAGGCAGGAGACTGACGA-3'; *Pdgfrb *promoter antisense, 5'-AGTCCCGGCTACCCTATCTGG-3'; *Mlf1 *promoter sense, 5'-TGCCATAGCAGCCGAGCGAT-3'; *Mlf1 *promoter antisense, 5'-GCTTGACGCAGGCCGTTTC-3'.

### RNA purification and RT-PCR

RNA was prepared using the Trizol reagent (GIBCO-BRL, Invitrogen Corp., Carlsbad, CA, USA). RT reactions were performed with Moloney murine leukemia virus from Finnzymes (Finnzymes, Espoo, Finland). SYBR Green I and Opticon monitor system from MJ Research (MJ Research, Waltham, MA USA) were used for real-time PCR reactions. Primers used for RT-PCR reactions were: *Nanog *sense, 5'-CGCTGCTCCGCTCCATAACT-3'; *Nanog *antisense, 5'-GCGCATGGCTTTCCCTAGTG-3'; *Nr0b1 *sense, 5'-CTGGTGTGCAGCGTCTGA-3'; *Nr0b1 *antisense, 5'-GTGTTGGTCTCCGGATCTC-3'; *Sall1 *sense, 5'-AGTTCTCCCAGGAGGCGAGGTG-3'; *Sall1 *antisense, 5'-GGTTGGCAGATGTTCGTAAAGT-3'; *Hoxa1 *sense, 5'-GGTCAACCCAACGCAGTG-3; *Hoxa1 *antisense, 5'-TGCTTCATGCGGCGATT-3'; *Pdgfrβ *sense, 5'-GACTACCTGCACCGGAACA-3'; *Pdgfrβ *antisense, 5'-GGGACTCAATGTCTGCGTATT-3'; *Mlf1 *sense, 5'-GAACCCATAATCGTCGAG-3'; *Mlf1 *antisense, 3'-CTTCGGGTTTGAGTTGAG-3'; *Sox17 *sense, 5'-CTCTGCCCTGCCGGGATGG-3'; *Sox17 *antisense, 5'-AATGTCGGGGTAGTTGCAATA-3'; Brachyury sense, 5'-GCGAGCTGGGTGGATGTAGA-3'; Brachyury antisense, 5'-CAAGGCGGCACAAGACTAAGTC-3; *Mash1 *sense, 5'-CCACCATCTCCCCCAACTA-3'; *Mash1 *antisense, 5'-CTGGGCTAAGAGGGTCGTAGG-3'; *Hprt *sense, 5'-CTCCTCAGACCGCTTTTTG-3'; *Hprt *antisense, 5'-TCCTCGGCATAATGATTAGG-3'.

### Protein and histone extracts

CGR8 cell extracts were prepared using a lysis buffer containing 50 mM Tris 8.0, 170 mM NaCl, 50 mM NaF, 0.5% NP-40, 1 mM PMSF. For histone extraction, cells were harvested, washed three times with ice-cold PBS supplemented with 5 mM Na butyrate and lysed with a Triton extraction buffer (PBS supplemented with 0.5% Triton, 2 mM PMSF, 0.02% NaN3) at a cell density of 10^7 ^cells/ml. The lysate was centrifuged at 2,000 rpm for 10 minutes at 4°C, and the pellet was washed twice with a half Triton extraction buffer volume and then resuspended in 0.2 N HCl at a cell density of 4 × 10^7 ^cells/ml. Histones were acid extracted overnight at 4°C. The next day samples were centrifuged at 2,000 rpm for 10 minutes at 4°C and the supernatant was dialyzed against CH_3_COOH and H_2_O. Samples were kept at -80°C with 20% glycerol.

### Microarray hybridization

Total RNA from ES cells was isolated using the RNeasy Mini Kit from QIAGEN (QIAGEN GmbH, Hilden, Germany) and treated with RNase-free DNase I (5 U/100 μg of nucleic acids, Sigma). Biotinylated cRNA was prepared according to the standard Affymetrix protocol. In brief, double-stranded cDNA was synthesized from 10 μg total RNA using the SuperScript Choice System from Invitrogen and the Affymetrix T7-(dT)_24 _primer, which contains a T7 RNA polymerase promoter attached to a poly-dT sequence. Following a phenol/chloroform extraction and ethanol precipitation, the cDNA was transcribed into biotin-labeled cRNA using the Retic Lysate IVT™ kit (Ambion Inc., Woodward Austin, TX, USA). cRNA products were purified using the RNeasy kit (QIAGEN) and fragmented to an average size of 30-50 bases according to Affymetrix recommendations. Fragmented cRNA (15 μg) was used to hybridize the Mouse Genome 430 2.0 Array for 16 h at 45°C. The arrays were washed and stained in the Affymetrix Fluidics Station 400 and scanned using the Hewlett-Packard GeneArray Scanner G2500A.

### Microarray data analysis

The image data were analyzed with the GeneChip^® ^Operating Software (GCOS) using Affymetrix default analysis settings. After a quality control test, arrays were normalized by log scale robust multi-array analysis (RMA) [44].

A parametric ANOVA (F-test) was performed. The false discovery rate of the resulting test-set was calculated using the Benjamini Hochberg procedure [45]. An f-test *p*-value < 10^-2 ^corresponding to a false discovery rate of 4.066 × 10^-2 ^and a fold change > 2 was used in order to select transcripts appearing in Additional data file 2 (792 down-regulated and 1,376 up-regulated transcripts). For the hierarchical clustering, a f-test *p*-value < 10^-3^, corresponding to a false discovery rate of 7.833 × 10^-3 ^and a fold change > 4 between the control and TSA treatment at 6 h or 12 h was used to identify and restrict the number of differentially expressed genes (fold change > 4, 458 probe sets). We then performed hierarchical clustering of the above 458 probesets to identify genes that respond similarly to the various experimental conditions. The cluster analysis was done using cluster version 2.11 [46] applying mean-centering and normalization of genes and arrays before average linkage clustering with uncentered correlation. The expression profiles of differentially expressed transcripts were discriminated into four groups according to the expression profile of the hierarchical clustering.

Functional annotation of transcripts differentially expressed ≥ 4-fold was done using the Database for Annotation, Visualization, and Integrated Discovery (DAVID) [31] to obtain Gene Ontology annotations for the category of 'biological process'. Transcript redundancies were removed based on statistical analysis and PCR validation data.

## Abbreviations

H3K27, Histone 3 lysine 27, H3K4, Histone 3, lysine 4; ALP, alkaline phosphatase; ChIP, chromatin immunoprecipitation; EB, embryoid body; ES, embryonic stem; ES^re^, ES released from TSA; PBS, phosphate-buffered saline; TSA, trichostatin A.

## Authors' contributions

EK performed experiments, analyzed data, produced figures and tables and contributed to the writing of the manuscript. HS, OH performed and NH guided the Affymetrix microarray analysis. AKH contributed to data analysis and manuscript organization. AK designed experiments, analyzed data and wrote the manuscript. All authors had the opportunity to discuss the results and comment on the manuscript.

## Additional data files

The following additional data are available with the online version of this paper. Additional data file 1 includes supplemental materials and methods, and Figures S1-S7. Additional data file 2 lists gene probesets that show ≥ 2-fold change in their mRNA levels after treatment of mouse ES cells with 50 nM of TSA for 6 and 12 h. Additional data file 3 lists gene probesets that show ≥ 4-fold change in their mRNA levels after treatment of mouse ES cells with 50 nM of TSA for 6 and 12 h, categorized according to their kinetics. Additional data file 4 lists the functional annotation of gene probesets of subcluster 1a based on biological process. Additional data file 5 lists the functional annotation of gene probesets of subcluster 1b based on biological process. Additional data file 6 lists the functional annotation of gene probesets of subcluster 2a based on biological process. Additional data file 7 lists the functional annotation of gene probesets of subcluster 2b based on biological process.

## Supplementary Material

Additional data file 1 Figure S1 shows *Nanog *mRNA levels after treatment of mouse ES cells with 10, 20 and 50 nM TSA for 6 and 12 h and Oct4 and Sox2 protein levels after treatment of mouse ES cells with 50 nM TSA for 6 and 12 h. Figure S2 shows *Oct4*, *Sox2 *and *Zfp42 *mRNA levels after treatment of mouse ES cells with 50 nM of TSA for 6 and 12 h and further release from TSA for 6 and 12 h. Figure S3 shows mRNA levels of 20 genes after treatment of mouse ES cells with 50 nM TSA for 6 and 12 h, as validation of the microarray experiment. Figure S4 shows a hierarchical clustering of gene probesets that show ≥ 4-fold change in their mRNA levels after treatment of mouse ES cells with 50 nM of TSA for 6 and 12 h. Figure S5 shows FACS analysis of mouse ES cells stained for Nanog protein after treatment with 50 nM TSA for 12 h and further release from TSA for 12 h. Figure S6 shows *Nanog*, *Nr0b1 *and *Sall1 *mRNA levels after treatment of mouse ES cells with 50 nM TSA for 1, 2, 4, 6 and 12 h. Figure S7 shows HDAC1 levels on the *Nanog *promoter after treatment of mouse ES cells with 50 nM TSA for 6 and 12 h. Click here for file

Additional data file 2 Gene probesets that show ≥ 2-fold change in their mRNA levels after treatment of mouse ES cells with 50 nM of TSA for 6 and 12 h. Click here for file

Additional data file 3 Gene probesets that show ≥ 4-fold change in their mRNA levels after treatment of mouse ES cells with 50 nM of TSA for 6 and 12 h, categorized according to their kinetics. Click here for file

Additional data file 4 Functional annotation of gene probesets of subcluster 1a based on biological process. Click here for file

Additional data file 5 Functional annotation of gene probesets of subcluster 1b based on biological process. Click here for file

Additional data file 6 Functional annotation of gene probesets of subcluster 2a based on biological process. Click here for file

Additional data file 7 Functional annotation of gene probesets of subcluster 2b based on biological process. Click here for file
