# Supplementary material for: Histone deacetylase inhibition accelerates the early events of stem cell differentiation: transcriptomic and epigenetic analysis
Source: Genome Biol. 2008 Apr 4;9(4):R65. doi: 10.1186/gb-2008-9-4-r65 (PMC2643936; doi:10.1186/gb-2008-9-4-r65)
Supplement: Additional data file 1 — Figure S1 shows Nanog mRNA levels after treatment of mouse ES cells with 10, 20 and 50 nM TSA for 6 and 12 h and Oct4 and Sox2 protein levels after treatment of mouse ES cells with 50 nM TSA for 6 and 12 h. Figure S2 shows Oct4, Sox2 and Zfp42 mRNA levels after treatment of mouse ES cells with 50 nM of TSA for 6 and 12 h and further release from TSA for 6 and 12 h. Figure S3 shows mRNA levels of 20 genes after treatment of mouse ES cells with 50 nM TSA for 6 and 12 h, as validation of the microarray experiment. Figure S4 shows a hierarchical clustering of gene probesets that show ≥ 4-fold change in their mRNA levels after treatment of mouse ES cells with 50 nM of TSA for 6 and 12 h. Figure S5 shows FACS analysis of mouse ES cells stained for Nanog protein after treatment with 50 nM TSA for 12 h and further release from TSA for 12 h. Figure S6 shows Nanog, Nr0b1 and Sall1 mRNA levels after treatment of mouse ES cells with 50 nM TSA for 1, 2, 4, 6 and 12 h. Figure S7 shows HDAC1 levels on the Nanog promoter after treatment of mouse ES cells with 50 nM TSA for 6 and 12 h. [file gb-2008-9-4-r65-S1.pdf]

SUPPLEMENTAL INFORMATION

Materials and methods

Antibodies

Antibodies used for western blot were from Santa Cruz (Oct4) and Abcam (Sox2) and for ChIP assay antibody used was from Upstate (HDAC1).

FACS analysis

Cell cytometry was performed using a FACSCalibur (Becton /Dickinson) instrument.

Primer sets

Oct4 sense : 5’- ACCAGGGTCTCCGATTTGCAT-3'

Oct4 antisense : 5’- CCCTGGGCGTTCTCTTTGGAA-3'

Sox2 sense : 5'-AGCTCGCAGACCTACATGA-3'

Sox2 antisense : 5'-TGGCCTCGGACTTGAC-3'

Rex1 sense : 5'-TCCTCAAGCCGGGTGCAA-3'

Rex1 antisense : 5'-CGGTGTGGATGCGGATATGGG-3’

Gdf3 sense: 5’- AGGAACTTCTGCCACCGTCA-3'

Gdf3 antisense: 5’- GCATGGAGATGGGCGAGAG-3'

Klf2 sense: 5’- ACACCAACTGCGGCAAGAC-3'

Klf2 antisense: 5’- TTTCGGTAGTGGCGGGTAAG-3'

Nupr1 sense: 5’- GGCATAATGGCCACCTTG-3’

Nupr1 antisense: 5’- CTCAGCGCCAGGCTTTTTTC-3’

Sall4 sense: 5’- ATGGAAGCCGAGGAGGTC-3'

Sall4 antisense: 5’- CAACACAGAGAAGCCCAGAGA-3'

Lefty2 sense: 5’- AGCACGCGACCGCTCCC-3'

Lefty2 antisense: 5’- CGCTGCTCCATTCCGAACAC-3'

Pcaf sense: 5’- GCCCTTGCTGCTCACGTTTC-3'

Pcaf antisense: 5'- GCTGGAAATGGCGGCTTC-3'

Gli2 sense: 5'- GCCGAGGTGGTCATCTACGA-3'

Gli2 antisense: 5'- GCGGCACACGAACTCCTTCT-3'

Egr1 sense: 5'-GGAGCCGAGCGAACAACCCTAT-3'

Egr1 antisense: 5'- TGGGAGGCAACCGAGTCGTTT-3'

Idb2 sense: 5'- CGACCCGATGAGTCTGCTCT-3'

Idb2 antisense: 5'- CCTGGACGCCTGGTTCTGT-3'

H1f0 sense: 5'- AACGCCGACTCCCAGA-3'

H1f0 antisense: 5'- GGCCACTTTCTTGACTTCCTT-3'

Fos sense: 5'- GGTTTCAACGCCGACTACGAG-3'

Fos antisense: 5'- AGGTCTGGGCTGGTGGAGAT-3'

Creg1 sense: 5'- CGGACATCATCTCAATCAG-3'

Creg1 antisense: 5'- CACAGTTCCCGACATCATTA-3'

Hspa1a sense: 5'- CTGGAGCCCGTGGAGAA-3'

Hspa1a antisense: 5'- GCCCGTTGAAGAAGTCCTG-3'

SUPPLEMENTAL FIGURE LEGENDS

Fig. S1. A. Nanog mRNA levels. ES cells were treated with 10, 20 and 50 nM TSA for 6 and 12 hours. Nanog mRNA levels were measured with RT-PCR analysis and were normalised to Gapdh. **B. Protein expression of Oct4 and Sox2.** Protein levels of Oct4 and Sox2 after treatment of ES cells with 50 nM TSA for 6 and 12 hours.

Fig. S2. mRNA levels of Oct4, Sox2 and Rex1. RT-PCR analysis was performed for the indicated genes after treatment of ES cells with 50nM TSA for 6 and 12 hours, removal of TSA and additional cultivation for 6 and 12 hours.

Fig. S3. Validation of microarray results for selected genes. RT-PCR analysis was performed for the indicated genes in control and TSA treated ES cells.

Fig. S4. Hierarchical clustering of transcripts differentially expressed ≥4 fold after 6 or 12hrs of TSA treatment. Numbers 1,2, and 3 represent three biological replicates of the experiment.

Fig. S5. Flow cytometric analysis of Nanog expressing cells. Nanog levels of untreated (A-control), TSA treated for 12 hrs (B-12 hrs TSA) and cells released for 12 hrs following a 12 hrs TSA treatment (C-12 hrs TSA +12 hrs removal) were analysed by intracellular staining for Nanog and flow cytometry using a rabbit anti-Nanog primary antibody (A,B,C) or a non specific antibody (D-NS antibody). Plots show the fluorescence intensity distribution, the mean fluorescent intensity (MFI) and percentage of cells above (M2) or below (M1) the threshold determined by the staining with the NS antibody.

Fig. S6. Kinetics of genes suppressed by TSA. RT-PCR analysis was performed for Nanog, Nr0b1 and Sall1 mRNA levels during TSA treatment.

Fig. S7. HDAC1 recruitment on Nanog promoter. HDAC1 levels on Nanog promoter were estimated using ChIP assay and real time PCR.

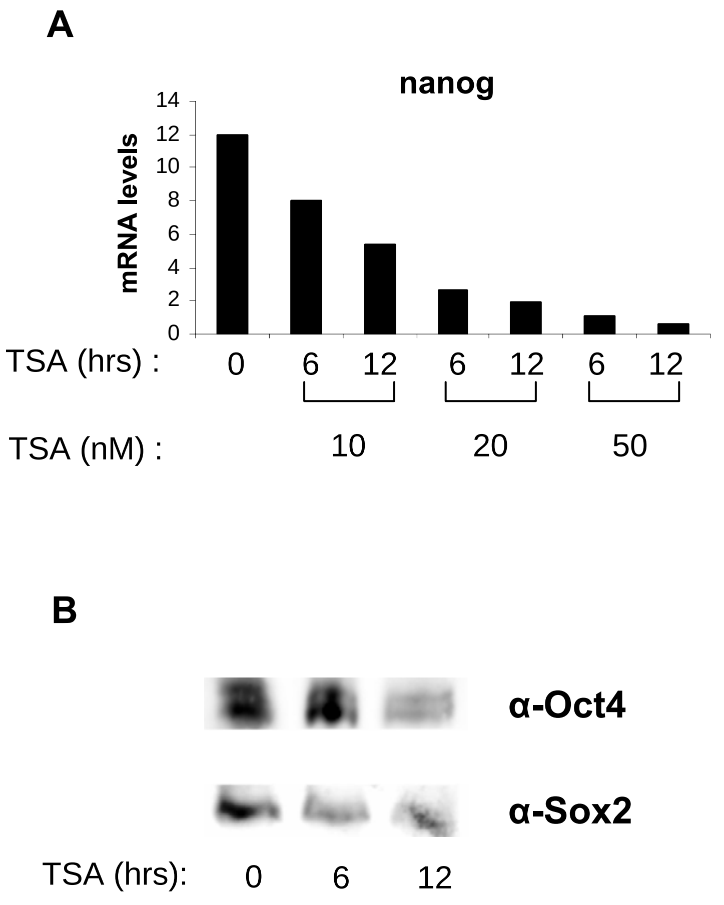
A
nanog
mRNA levels
14
12
10
8
6
4
2
0
TSA (hrs) :
0
6
12
6
12
6
12
TSA (nM) :
10
20
50
B
α-Oct4
α-Sox2
TSA (hrs):
0
6
12

Fig.S1

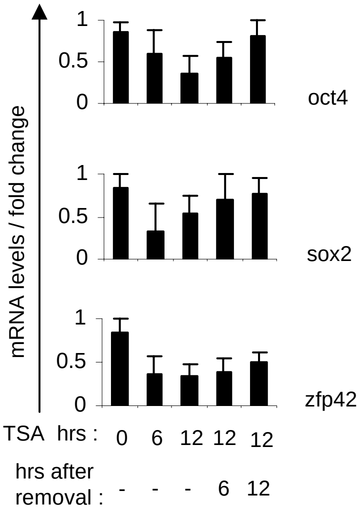
mRNA levels / fold change
1
0.5
0
oct4
sox2
zfp42
TSA hrs :
0
6
12
12
12
hrs after
removal :
-
-
-
6
12

Fig.S2

Microarray validation

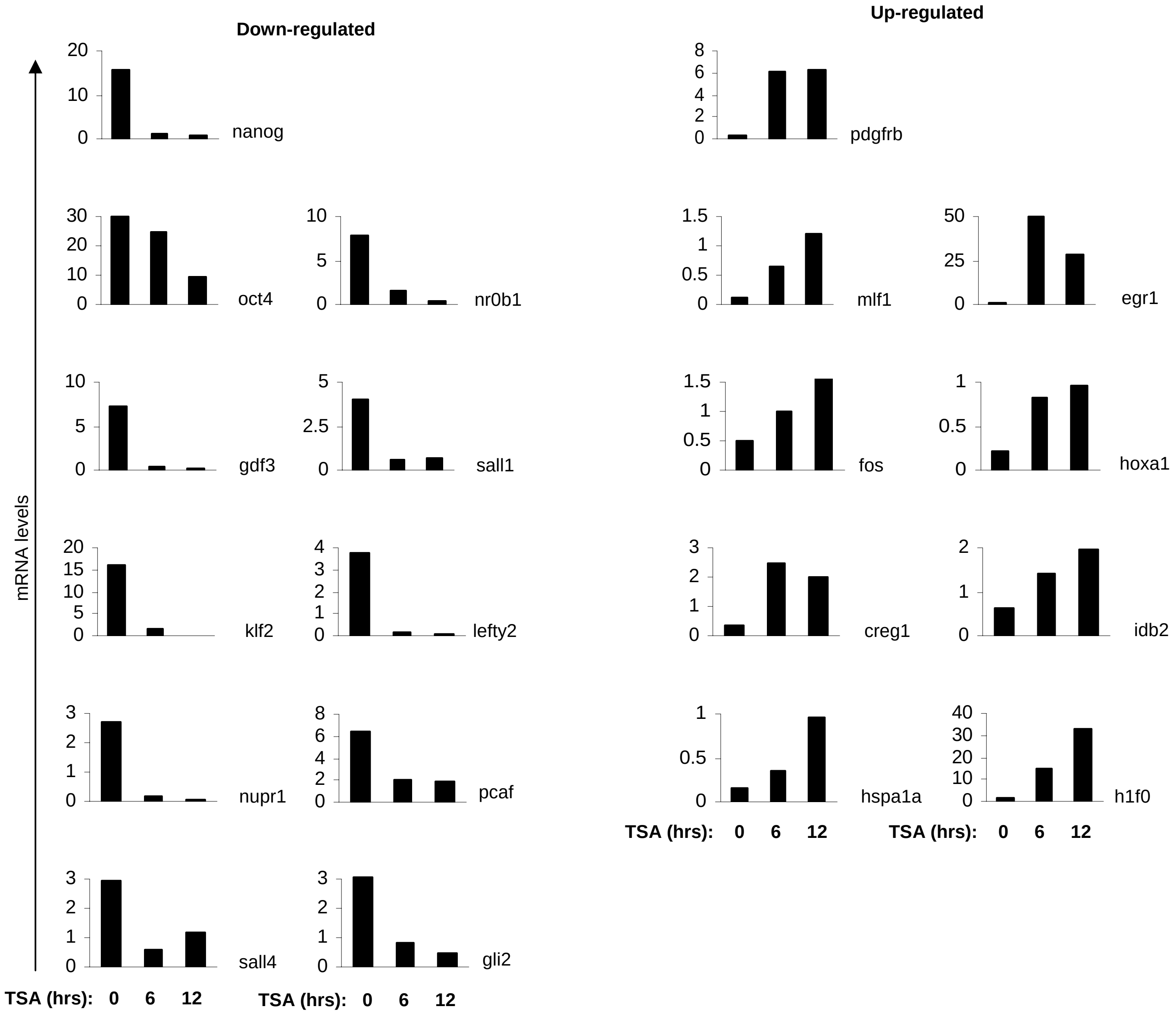

Fig.S3

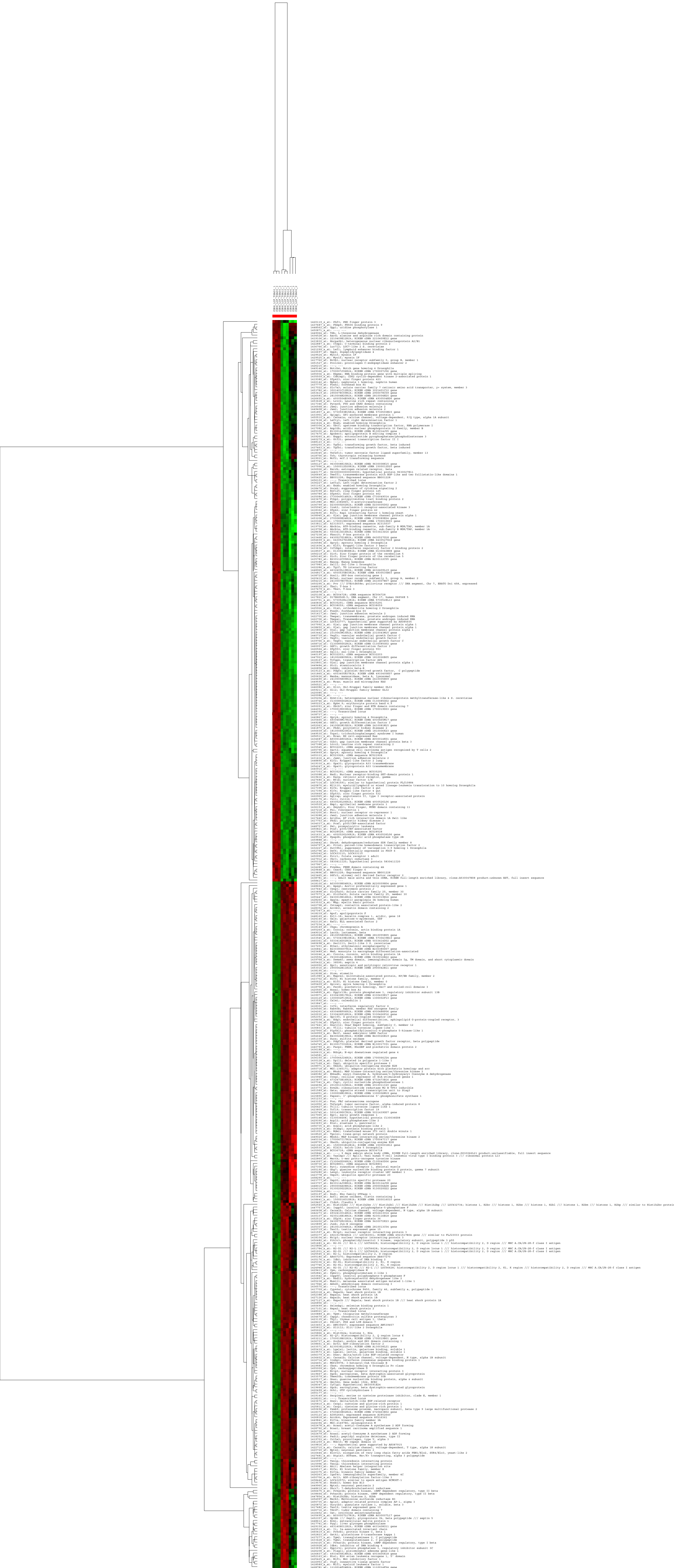

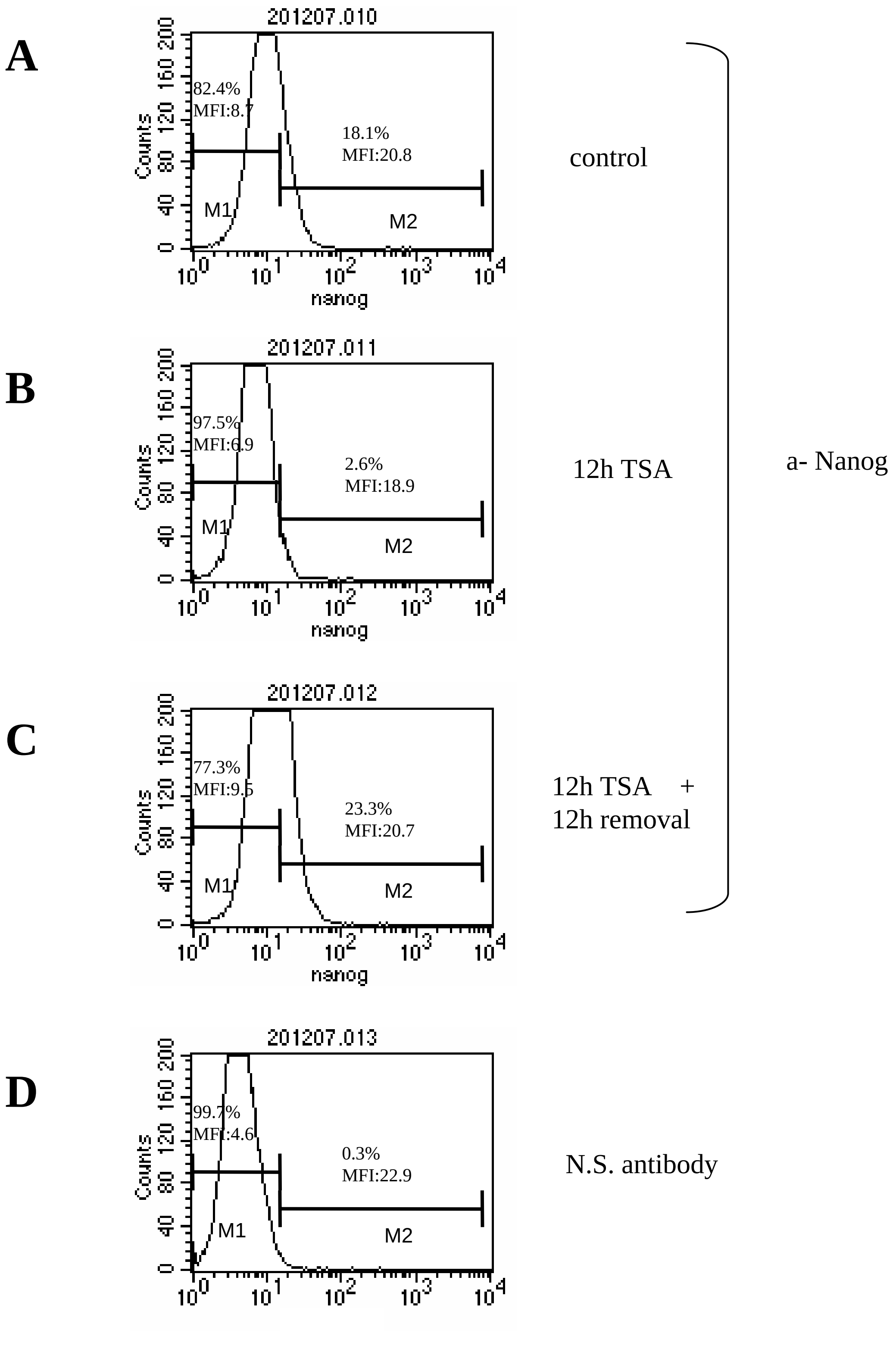
A
201207.010
82.4%
MFI:8.7
18.1%
MFI:20.8
M1
M2
Counts
nanog
control
B
201207.011
97.5%
MFI:6.9
2.6%
MFI:18.9
M1
M2
Counts
nanog
12h TSA
a- Nanog
C
201207.012
77.3%
MFI:9.5
23.3%
MFI:20.7
M1
M2
Counts
nanog
12h TSA +
12h removal
D
201207.013
99.7%
MFI:4.6
0.3%
MFI:22.9
M1
M2
Counts
N.S. antibody

Fig.S5

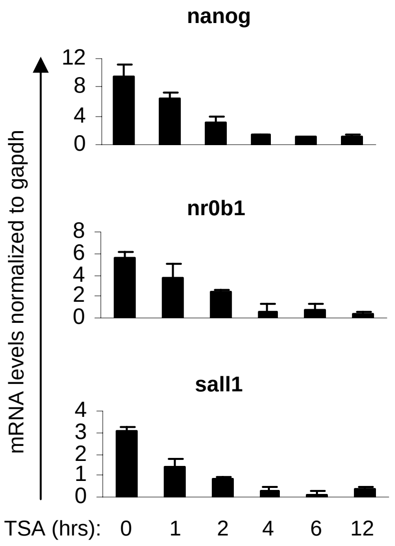
nanog
12
8
4
0
nr0b1
8
6
4
2
0
sall1
4
3
2
1
0
mRNA levels normalized to gapdh
TSA (hrs): 0 1 2 4 6 12

Fig.S6

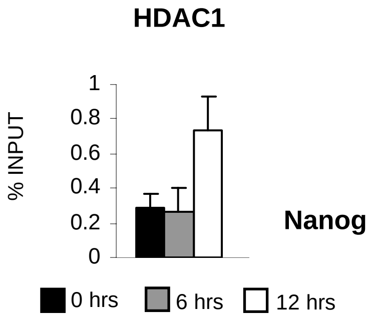

Fig.S7
